# Design and development of genetically encoded fluorescent sensors to monitor intracellular chemical and physical parameters

**DOI:** 10.1007/s12551-016-0195-9

**Published:** 2016-04-29

**Authors:** Arno Germond, Hideaki Fujita, Taro Ichimura, Tomonobu M. Watanabe

**Affiliations:** 1 Laboratory for Comprehensive Bioimaging, RIKEN Quantitative Biology Center (QBiC), 6-2-3 Furuedai, Suita, Osaka 565-0874 Japan; 2 WPI Immunology Frontier Research Center, Osaka University, 1-3 Yamadaoka, Suita, Osaka 565-0871 Japan

**Keywords:** Fluorescent protein, Green fluorescent protein, Genetically encoded, Intracellular sensor, Fluorescent measurement, Probe design

## Abstract

Over the past decades many researchers have made major contributions towards the development of genetically encoded (GE) fluorescent sensors derived from fluorescent proteins. GE sensors are now used to study biological phenomena by facilitating the measurement of biochemical behaviors at various scales, ranging from single molecules to single cells or even whole animals. Here, we review the historical development of GE fluorescent sensors and report on their current status. We specifically focus on the development strategies of the GE sensors used for measuring pH, ion concentrations (e.g., chloride and calcium), redox indicators, membrane potential, temperature, pressure, and molecular crowding. We demonstrate that these fluroescent protein-based sensors have a shared history of concepts and development strategies, and we highlight the most original concepts used to date. We believe that the understanding and application of these various concepts will pave the road for the development of future GE sensors and lead to new breakthroughs in bioimaging.

## Introduction

The development of engineered fluorescent proteins (FPs) started with the discovery by Shimomura and colleagues of the green fluorescent protein (GFP), which causes the natural green bioluminescence of the Pacific Ocean jellyfish *Aequorea victoria* (Shimomura et al. 1962). However, the value of GFP was only fully realized some 30 years later when Chalfie and co-workers isolated GFP cDNA and then expressed its fluorescent protein product in bacteria and the nematode *Caenorhabditis elegans* (Prasher et al. 1992). Subsequent years have witnessed improvements in the fluorescence of GFP and its stabilization by genetic manipulation (Cubitt et al. 1995). In addition, color variants exhibiting various emission spectra have been engineered by means of direct mutagenesis (Tsien 1998; Nifosí et al. 2007) and/or isolation from different cnidarians, such as button polyps (*Zoanthus sp*.), mushroom coral (*Discosoma sp*.), snakelocks Anemone (*Anemonia Sulcata*), among others (Chudakov 2010). Various GFP-like proteins have also been endowed with functions other than fluorescence, and color variants have been developed that have found numerous applications in in vivo imaging (Chudakov 2010). Nowadays, FPs are extensively used in the fields of biochemistry, biotechnology, and cell and development biology.

The use of chemical organic dyes for measuring the behavior of intracellular environments has been limited by the difficulty to control the cell permeabilization needed for labeling. To counter these limitations, genetically encoded (GE) sensors have been developed from FPs which enable visualization and quantification of various intracellular physiological events in living cells, tissues, and/or whole organisms. For example, GE sensors allow researchers to observe and measure the dynamic behaviors and/or expressions of target proteins, with the functions and conformational state of these proteins not only becoming optically visible in living specimens, but also being optically activated or deactivated (Aoki et al. 2013; Sample et al. 2014). GE sensors have also been developed to probe the variations of pH or changes in the chemical concentrations of specific ions.

**Fig. 1:**
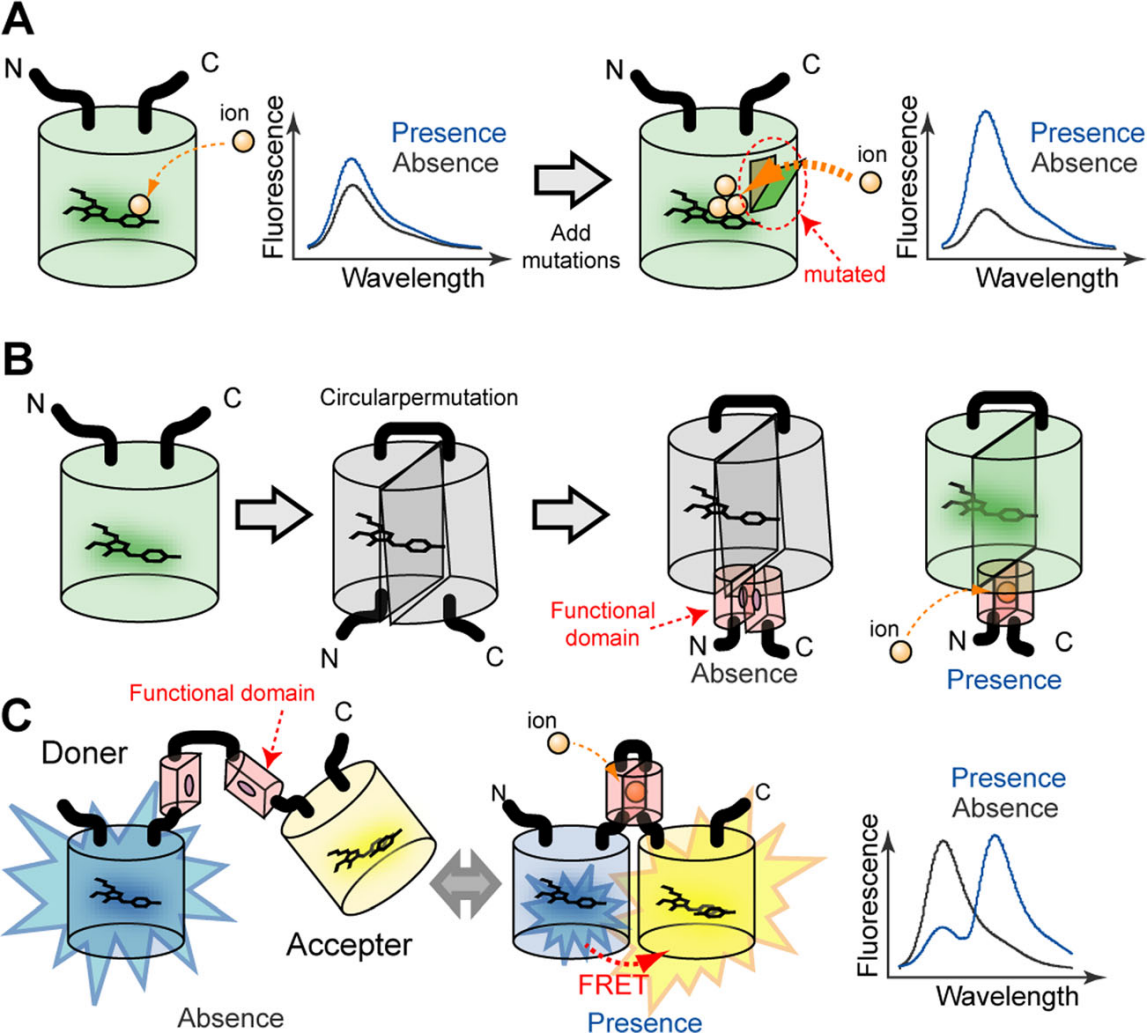
Schematic drawing of the three main general strategies to develop genetically encoded (GE) sensors from fluorescent proteins (FPs). **a** Strategy 1: Employment of the intrinsic sensitivity of FPs to certain conditions of the solution environment. Researchers investigate the most effective mutations to enhance the sensitivity. **b** Strategy 2: Use of a circular-permutation technique in which the functional domain is fused with the circularly permutated FP, thereby converting the ion binding to fluorescence emission. **c** Strategy 3: Use of the Förster resonance energy transfer (*FRET*) technology. The functional domain between the donor and the accepter converts the ion binding to the fluorescence spectrum

The design and development of GE sensors is now leading a major evolution in the bioimaging of single molecules, cells, tissues, and whole organisms. For example, neuroscience is a research field where GE sensors are strictly applied, and even the millisecond-reaction spike potentials are now be detectable under a fluorescence microscope (Knöpfel 2012). In this review, we introduce and discuss the three major development strategies and the various design concepts used to design GE sensors from FPs. The first strategy is to exploit the intrinsic sensitivity of FPs to certain conditions of the solution environment (Fig. 1a). To this end, effective mutations are added to the periphery of the chromophore or at specific sites in order to selectively enhance the sensitivity of an FP. For example, even though FPs are often undesirably sensitive to various conditions, such as pH, which may on occasionally cause considerable problems in imaging (Patterson et al. 1997; Wachter and Remington 1999), from a different point of view the sensitivity of FPs to pH can be used as a strategy to develop GE pH sensors. The second strategy is based on the use of a circular-permutation technique (Fig. 1b) (Baird et al. 1999). Circular permutations, which can occur naturally or, alternatively, be engineered, produce a protein whose amino acids are in a different sequence; this different structure may improve some property of the protein. Because of this disruption to the structure of the original protein, circular-permutated FPs (cpFPs) cannot generate the fluorescence of the original protein. This property can be exploited by inserting a functional domain into the cpFP which, in a specific condition of the solution environment, can restore the fluorescence activity due to a conformational change of the inserted domain. The third strategy is the use of Förster resonance energy transfer (FRET) technology, which involves the distance-dependent energy transfer from a FP (donor) excited at a particular wavelength to another FP (acceptor) (Fig. 1c) (Förster 1948). Normally, a functional domain is inserted between the donor FP and the acceptor FP. The structural changes caused by the solution conditions are observed via changes in the fluorescence spectrum that is derived from the change in FRET efficiency which in turn is dependent on the distance between the donor and the acceptor.

By using and/or combining these strategies, biologists have been able to engineer a large variety of GE sensors. In this review, we are particularly interested in the sensors used to measure intracellular chemical or physical parameters. In addition, we introduce and discuss the most recent developments in GE sensors which were developed to measure the state of water in a cell, including temperature, pressure, and molecular crowding. To allow readers to easily refer to the category of sensors which most interest them, we have organized this review such that each section focuses on a different type of GE sensor.

## Intracellular pH sensors

The regulation and homeostasis of pH are crucial for the viability of any living cell. Numerous intracellular biological functions are highly dependent on the ability of cells to regulate intracellular pH within the cell and its subcellular compartments. For example, most organelles of eukaryotic cells, such as mitochondria, exhibit a specific pH value. To monitor the pH of individual subcellular compartments in mammalian cells, researchers judged it desirable to develop genetically encoded pH sensors with high fluorescence in the range of pH 6.0–8.0. Ward et al. (1980) observed that the fluorescence of the wild-type GFP derived from *Aequorea victoria* is relatively stable in the biological pH range of 6.0–8.0. The chromophore of the *Aequorea* GFP was found to have two alternative conformations depending on protonation states, corresponding to two absorbance spectral peaks at 395 nm in the protonated state and 475 nm in the deprotonated state (Heim et al. 1994; Brejc et al. 1997; Scharnagl et al. 1999). Although the excitation wavelength at 475 nm is visible, it is only a minor contributor to the fluorescence in wild-type *Aequorea* GFP. Two mutations, F64L and S65T, generated the first engineered version of *Aequorea* GFP, referred to as enhanced GFP (EGFP), which exhibited a higher fluorescence intensity at 475 nm (Heim et al. 1995). Interestingly, the fluorescence intensity of an EGFP when excited at 475 nm light in the deprotonated chromophore was shown to be directly related to the pH of the solution (Patterson et al. 1997). Verkman and colleagues took advantage of this enhanced pH dependency to observe intracellular pH variations via observed changes in the fluorescence intensity of the EGFP (Kneen et al. 1998). A yellow variant of EGFP, called EYFP (Ormö et al. 1996), showed an even larger pH sensitivity (pKa = 7.1) than the EGFP (pKa = 6.15) (Llopis et al. 1998). Tsien and colleagues fused an EYFP to an N-terminal domain of a galactosyltransferase or subunit IV of a cytochrome C oxidase and succeeded in detecting pH changes in the medial/trans-Golgi or the mitochondria within a single living cell (Llopis et al. 1998).

**Fig. 2:**
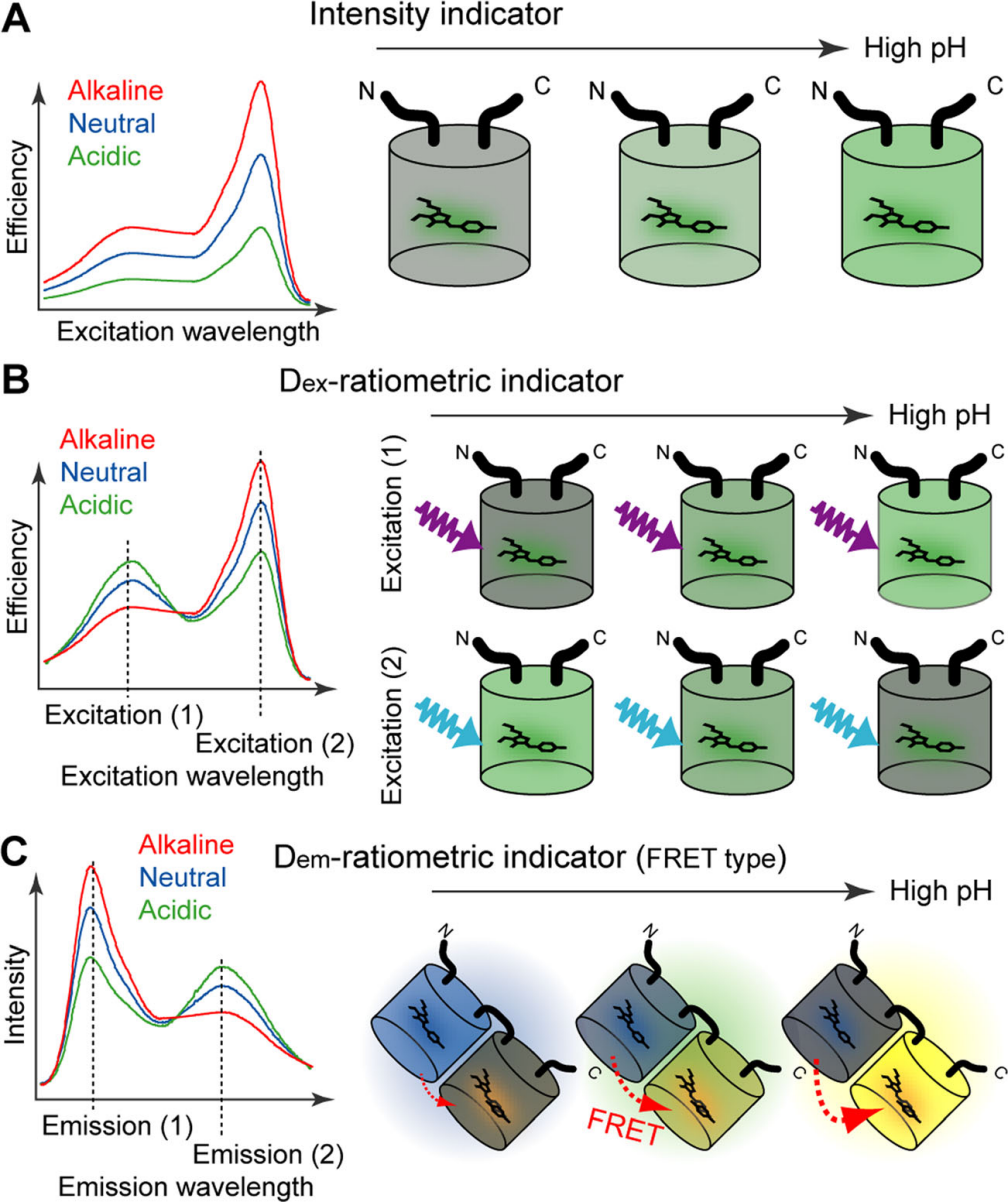
Schematic drawing of main three kinds of FP indicator. **a** Intensity indicator: the fluorescent intensity depends on the environment of the solution. **b** Dual excitation mode (*D*_*ex*_) ratiometric indicator: the excitation spectrum shows two peaks that respond in an antiparallel manner to the solution environment; two distinct excitations are needed. (**b**) Dual emission mode (*D*_*em*_) ratiometric indicator: the emission spectrum shows two peaks that respond in an antiparallel manner to the solution environment

Fluorescence intensity depends not only on the pH value, but also on other factors, including, for example, the expression level of the gene and photobleaching. Hence, the absolute pH value cannot be estimated from changes in the fluorescence. To circumvent these limitations and also be able to measure the absolute pH, researchers developed ratiometric pH indicators with dual excitation (D_ex_) and dual emission mode (D_em_) mode. These GE sensors exhibit bimodal excitation and/or emission spectra with pH-dependent changes in excitation/emission and, consequently, they can be used to quantify the absolute pH value from the spectral properties (Fig. 2). Here, we present the example of a D_ex_-ratiometic pH sensor, termed ratiometric pHluorin, that was developed to measure absolute pH (Miesenböck et al. 1998). The variant contains a cluster of mutations that direct pH sensitivity. The mutations were chosen among seven residues, namely, Gln-94th, Arg-96th, His-148th, Ile-167th, Thr-203th, Ser-205th, and Glu-222th, in an *Aequorea* wild-type GFP to alter the environment near the chromophore (Heim and Tsien 1996; Ormö et al. 1996; Yang et al. 1996; Brejc et al. 1997). The S202H mutation, which replaces the Ser-202th with a histidine, causes the antiparallel reaction at excitation peaks of 395 nm and 475 nm (NOT absorbance). Upon a pH change, the excitation efficiency at 395 nm increases and the excitation intensity at 475 nm decreases when the pH is shifting from 5.5 to 7.5. This means that the pH value can be estimated by calculating the ratio of the fluorescence intensities at excitation peaks of 395 nm and 475 nm, respectively. The pH sensitivity and the fluorescence intensity of pHluorin were further enhanced by the additional mutations of F64L, R80Q, D132E, and G175S, which endowed the protein with a ratiometric property (Mahon 2011).

Red GE pH sensors were developed by adding mutations into FPs isolated from *Discosoma* sp. (Shaner et al. 2004). The mFruit series of GE pH sensors is composed of mutated variants that show pH sensitivity, including a number of variants with a relatively higher pH sensitivity, such as mBanana, mOrange, mOrange2, and mApple (pKa = ~6.5) (Shaner et al. 2008; Johnson et al. 2009). Additional mutations were subsequently developed which still further enhanced pH sensitivities, including mNectarine, whose pKa of 6.9 is based on mTangerine with a pKa of 5.7 (Johnson et al. 2009). The most pH sensitive red FP developed to date is pHTomato, whose pKa of 7.8 is based on mStrawberry (Li and Tsien 2012) in which six amino acids were mutated by DNA shuffling and semi-random mutagenesis (Tsutsui et al. 2005). Although these variants satisfy the need for higher pH sensitivity, as indicated by their pKa value, the degree of change is approximately three- and sixfold for pHTomato and mNectarine, respectively, whose values are in turn approximately 50-fold smaller than that of pHluorin. To counter this limitation, an orange pH sensor named pHoran4 and a red pH sensor named pHuji were optimized by focusing on the fluorescence change in the pH range of 5.5–7.5 (Shen et al. 2014).

A D_ex_-ratiometric pH sensor of a red FP was also developed and named pHRed; this sensor is based on a unique FP, mKeima, derived from *Montipora* sp. (Tantama et al. 2011). While the GFP-like violet-colored chromoprotein in *Montipora* sp. does not fluoresce in nature, appropriate multiple mutations made the pHRed fluorescent and monomerized, resulting in a new type of FP with a long Stokes shift (Kogure et al. 2006). The multiple mutations reduced one excitation peak at 580 nm in the deprotonated state and maintained the other peak at 452 nm in the protonated, resulting in the long Stokes shift. In this process, one mutation in particular, S213A, was critical. The pHRed was obtained by the re-substitution of Ala-213th in mKeima for serine. This successful process demonstrated that a ratiometric pH sensor could be developed by altering the transition in protonated–deprotonated states of the phenolic hydroxyl moiety of the chromophore. In terms of biology, pHRed exhibits a pH-dependent fluorescence lifetime that makes it suitable for fluorescence lifetime imaging microscopy (FLIM).

The effective pH sensitivity range of the above-mentioned pH sensors were subsequently adjusted to pH 6–8 for applications in mammalian cells. However, these sensors cannot be applied to other biological studies, such as the monitoring of intracellular pH in plant cells. In plants, the control of pH in the cytosol and in the apoblast is an important process in the regulation of cell elongation, organ development, and nutrient transport. A GE pH sensor named pHusion has recently been designed specifically for pH measurements in the apoblast, where the pH fluctuates around a value of 5–6 (Gjetting et al. 2012). The pHusion pH sensor is a tandemly fused protein of two FPs, i.e., EGFP and monomeric red fluorescent protein 1 (mRFP1), with distinct emission peaks and pH sensitivities: the pKa of EGFP is approximately 6.2 (Llopis et al. 1998) and that of mRFP1 is approximately 4.5 (Campbell et al. 2002). The fluorescence intensity of EGFP changes more than that of mRFP at higher pH; at low pH the reverse is true. The relationship between the fluorescence intensity of pHusion and the solution pH is that of a gentle titration curve in the range of pH 5–9 with the pKa of approximately 6.0, which is suitable for studies of both the apoblast and symplast. It should be noted that the dynamic range of the sensor’s sensitivity must be set at the appropriate value with respect to the subject.

## Intracellular chloride ion sensors

The chloride ion (Cl^-^) is one of the main anion species in the body and is involved in numerous cellular functions. For example, the amplitude of the inhibitory currents in the nervous system is determined by the intracellular concentration of Cl^-^. During the development of the various color variants of pH sensors of *Aequorea* GFP, researchers noted that the T203Y mutation enhances not only pH sensitivity, but also halide sensitivity (Wachter and Remington 1999; Arosio et al. 2007). The O_3_–C_3_ carbonyl bond in the chromophore of GFP variant S65T/T203Y (E^2^GFP) has two alternative conformations depending on chloride binding (Bizzarri et al. 2006; Arosio et al. 2007). By substituting a tyrosine for Thr-203th, the C_3_ of the chromophore and OH of Tyr-203th is bridged by the hydrogen bond via a water molecule. This results in the flipping of the O_3_–C_3_ carbonyl bond in the chromophore toward Tyr-203th, leading to a reduction of absorbance at 400 nm. When a chloride ion binds to Tyr-203th instead of water, the hydrogen bond bridging the C_3_ of the chromophore and OH of Tyr-203th becomes dissociated, and the O_3_–C_3_ conformation is flipped back to the normal conformation, causing the emergence of the absorbance at 400 nm.

A yellow variant of GFP, the widely used YFP, was generated by the T203Y and further mutations to a wild-type GFP, S65G, V68L, and S72A (Ormö et al. 1996). These mutations lead to the π–π stacking of the chromophore and the phenol ring of Tyr-203th, resulting in a red shift of the fluorescence and an enhancement of Cl^-^ sensitivity (Ormö et al. 1996). Another yellow variant named Citrene was developed to prevent the invasion of water molecules near the chromophore and also reduced the chloride sensitivity by the mutation of Q69M (Griesbeck et al. 2001). Venus, which is well known as a fast-maturating YFP mutant, also lacked Cl^-^ sensitivity (Nagai et al. 2002). On the other hand, the H148Q mutation was found to increase the affinity of Cl^-^ to the chromophore of YFP (Wachter and Remington 1999; Wachter et al. 2000; Jayaraman et al. 2000). By a random mutation based on the YFP–H148Q as a starting template, the further mutation of I152L was found to enhance the Cl^−^ sensitivity (Galietta et al. 2001). Most of the Cl^−^ sensory FPs sense the other halide ions. The V163S mutation to a YFP–H148Q increased the selectivity of the Cl^−^ sensitivity: he dissociation constant (*K*_d_) for Cl^−^ decreased from 197 to 62 mM while that for I^-^ increased from 20 to 107 mM (Galietta et al. 2001). Recently, a cell-free protein engineering method was used to screen the mutation that exhibited the highest Cl^-^ affinity and the dynamic range of YFP. The most effective mutation was the double mutation of Q69T and V163A, which achieved a selective Cl^-^ sensing with a *K*_d_ of about 20 mM (Grimley et al. 2013)

Clomeleon was the first GE D_em_-ratiometric Cl^-^ sensor obtained by fusing a YFP mutant (including H79R and L68V to enhance the Cl^-^ sensitivity) with a cyan variant insensitive to Cl^-^ (Kuner and Augustine 2000). Clomeleon is based on FRET: the YFP mutant is excited by the transfer of energy from the excited CFP (Figs. 1c, Fig. 2c, lower) which allows ratiometric measurement of absolute Cl^−^ concentrations. The CFP–YFP pair has been the most widely used FRET pair ever since (Felber et al. 2004). The Clomeleon undergoes the antiparallel changes of the CFP (approx. 480 nm) and the YFP (approx. 530 nm) fluorescence peaks by changing the Cl^-^ concentration in the solution due to the reduced excitation efficiency of the YFP mutant by the Cl^-^ binding. Though the *K*_d_ for Cl^-^ of the original Clomeleon is approximately 87–167 mM, which greatly exceeds the physiological range (approx. 3–60 mM), the replacement of the YFP–H79R–L68V in Clomeleon to a YFP–H148Q–I152L–V163S remarkably improves the *K*_d_ for Cl^−^ to 30 mM, which allows its use for physiological observation (Markova et al. 2008).

However, due to the relatively poor photostability of YFP derivatives, as well as to the low Cl^−^ sensitivity and the low pH susceptibility as a result of the modifications to YFP, there was the belief the YFP-based sensor was not optimal for precise dynamic and quantitative Cl^−^ measurements in vivo. Therefore, new improvements in GE YFP sensors were made. For example, a new FRET pair named SuperClomeleon (Grimley et al. 2013) was developed by shortening the linker between the CFP and the YFP mutant of the FRET pair and by adding the mutation S30R, which is known to stabilize the folding and enhance the brightness of *Aequorea* GFP variants (Pédelacq et al. 2006), in both of the CFP and the YFP mutants. These modifications not only resulted in improved Cl^-^ sensitivity, but also in a fourfold increase in the signal-to-noise ratio in ratiometric Cl^-^ measurements by comparison to the original GE D_em_-ratiometric Cl^-^ sensor Clomeleon. The new mutation was found to reduce pH sensitivity and enhance photostability in Cl^-^-sensitive YFP, achieving a Cl^-^ *K*_d_ of 14 mM and a pKa of 5.9 with a 15-fold longer bleach time constant (175 seconds) than YFP (Zhong et al. 2014).

## Intracellular calcium ion sensors

The calcium ion (Ca^2+^) has many essential roles in cellular signaling and biological function, such as in muscle contraction, apoptosis, neuronal transmission, among others. GE sensors were initially developed in an attempt to use non-invasive measurements of the Ca^2+^ dynamics (Koldenkova and Nagai 2013). The *Aequorea* GFP was found in a Ca^2+^-sensitive bioluminescent protein, aequorin, and the first GE Ca^2+^ sensor was the *Aequorea* aequorin itself (Shimomura et al. 1962). Currently the most famous Ca^2+^ sensory FP is perhaps Cameleon (NOT chameleon), developed by Tsien and coworkers (Miyawaki et al. 1997). The Cameleon is a FRET pair of CFP and YFP [or the blue GFP variant (BFP), and GFP] conjugated with calmodulin (CaM) and a CaM-binding peptide of myosin light chain kinase (M13) (see Fig. 1c) (Ikura et al. 1992). CaM and M13 are linked together with a flexible linker, a glycylglycine linker (Porumb et al. 1994). In the absence of Ca^2+^ in solution, FRET in the Cameleon does not occur because the CaM is unbound to M13 and the two FPs are far apart. When the CaM forms a complex with the M13 through the binding of Ca^2+^ to CaM, the CFP or BFP approaches the YFP or GFP so as to transfer the energy. The CaM in the Cameleon is tuned by a substitution, E104Q, to improve the affinity to Ca^2+^. The CFP–YFP pair have been subsequently improved as follows. While the first Cameleon was quite sensitive to pH (pKa = 6.9), the two substitutions of V68L and Q69K into YFP reduced the pH sensitivity of the Cameleon (pKa = 6.1) (Miyawaki et al. 1999). Here, it is useful to mention that the reduced pH sensitivity is particularly important for measurements of Ca^2+^ within specific organelles, such as the endosomal and lysosomal systems. Typically, pH-sensitive fluorescent protein gets quenched in acidic environments which has restrained its applications in vivo.

The replacement of the YFP in a Cameleon by a circular-permutated Venus (cpVenus) was found to increase the fluorescent stability and the Ca^2+^ sensitivity so that the contrast of intensity ratio was increased by approximately sixfold (Nagai et al. 2004). It is to be noted here that these authors did not completely monomerize both FPs, even though the *Aequorea* GFP variants tend to form a dimmer at high concentration due to weak dimerizing affinity (Yang et al. 1996; Phillips 1997). Because the FRET efficiency depends on the arrangement of two dipole moments of the donor and the acceptor, this weak dimerization of the FPs in a FRET pair helps in the aligning of the angles of the two dipoles (Vinkenborg et al. 2007). Further studies led to the development of a new Cameleon series, named the Cameleon–Nano series, which allows various dynamic ranges in Ca^2+^ measurements by controlling the length of the linker between CaM and M13 (Horikawa et al. 2010).

GCaMP (Nakai et al. 2001), GECO (Zhao et al. 2011), and Pericam (Nagai et al. 2001) are single FP-based Ca^2+^ sensors without FRET which have been developed with a common principle. In these Ca^2+^ sensors, the CaM and the M13 are fused to the N- and C- termini of the cpFP, respectively (see Fig. 1b). A cpFP lacks fluorescence because due to the instability of its structure. When Ca^2+^ binds to the CaM of these sensors, the conformation change of the CaM and the M13 stabilizes the structure of the cpFPs and restores the fluorescence. Red variants of the Ca^2+^ sensor were obtained later based on mCherry or mApple (Zhao et al. 2011; Carlson and Campbell 2013). The GECO series not only provides various color variants but also exhibits a long Stokes shift and ratiometric shift (Zhao et al. 2011). Alternatively, the Pericam series provides the opposite reaction in that the Ca^2+^ binding diminishes the fluorescence (Nagai et al. 2001). In some cases, the multi-colorizing aspect is more important than the ratiometric sensors in monitoring intracellular Ca^2+^ dynamics because the Ca^2+^ dynamics coordinates with other behaviors, such as changes in the membrane potential in neurons. There is also a Ca^2+^ sensor composed of two halves of a split FP instead of a cpFP and the CaM–M13 complex, based on the same strategy (Baird et al. 1999).

More recently, Saito and coworkers developed a chemiluminescent Ca^2+^ sensor which does not require the excitation illumination for Ca^2+^ monitoring (Saito et al. 2012). This achievement may become a milestone in the world of Ca^2+^ imaging. In fact, it has been quite difficult to observe the dynamic of Ca^2+^ behavior in organs, such as the brain, because the illumination light cannot penetrate deeply into the tissues of living organs. Future developments of such sensors may not only provide new tools for Ca^2+^ monitoring, but also lead to the appearance of other kinds of GE sensors. For more detailed information on the development of Ca^2+^ sensors, we strongly suggest that the reader refer to the excellent review of V. P. Koldenkova (Koldenkova and Nagai 2013).

## Sensors for other ions

During the development of Cameleon, it was shown that a key component for “sensitivity” is the conformational change of the CaM–M13 complex or the interactive binding of CaM and M13 with Ca^2+^ binding between a CFP and a YFP. This mechanism was intuitively understandable to the development of new strategies for other ions and macromolecules, such as Zn^2+^ (Qiao et al. 2006; Dittmer et al. 2009; Vinkenborg et al. 2009; Miranda et al. 2012), Mg^2+^ (Wegner et al. 2010; Lindenburg et al. 2013), Cu^+^ (Koay et al. 2013), inositol trisphosphate (IP3) (Tanimura et al. 2004; Sato et al. 2005a; Matsu-ura et al. 2006), cGMP (Sato et al. 2000; Honda et al. 2001; Nikolaev et al. 2006), cAMP (Zaccolo and Pozzan 2002; Nikolaev et al. 2004), ATP (Imamura et al. 2009), among others. We introduce here those sensors whose development was based on different strategies from those used to develop Cameleon.

The chromophore of FPs is enclosed in a β-barrel scaffold, and the properties of this scaffold depend on the protein matrix vicinity. Moreover, the chromophore tightens the β-barrel scaffold and plays a crucial role in its stability. By taking advantage of this characteristic, Vinkenborg et al. (2007) demonstrated that the dimerization characteristic of FPs can be controlled by altering the interface of the β-barrel. Interestingly, the wild-type GFP was found to weakly dimerize with β-barrels in solution when the two GFPs are brought into proximity (Prasher et al. 1992; Ormö et al. 1996; Yang et al. 1996; Phillips 1997). Jensen and colleagues succeeded in developing a FRET Zn^2+^ sensor based on the X-ray structure information of GFP (Jensen et al. 2001). These authors substituted the appropriate site at the dimerization interface with zinc-binding residues (cysteine and histidine) so that the CFP and the YFP of a CFP–YFP FRET pair was dimerized via the Zn^2+^ binding to those residues. The Tyr-39th and Ser-208th were selected and substituted for histidine (Y39H) and cysteine (S208C), respectively. Evers and colleagues subsequently optimized the distance between CFP and YFP by testing the linkers of various lengths and named this mutated CFP–YFP sensor ZinCh (Evers et al. 2007). The affinity of the ZinCh for Zn^2+^ is approximately 200 nM via the cysteine residues and approximately 90 μM via the histidine residues. The same group also found that the his-tag fusion at both termini increased both Zn^2+^ selectivity and sensitivity of ZinCh (Evers et al. 2008). However, cysteine and histidine are known to bind to other divalent metal ions, and unfortunately Cd^2+^ was detected with the ZinCh sensor, which exhibited a higher affinity with the cysteine residue than histidine residue (Evers et al. 2007).

The aforementioned ZinCh affinity for Cd^2+^ has enabled the development of new GE sensors to monitor intracellular heavy metal ions. For example, it has long been common knowledge that exposure to Cd^2+^ is harmful to human health due to the potential of heavy metal particle deposition in organs, such as the liver, which may compromise their functions (Nordberg 2009). GE Cd^2+^ sensors are expected to greatly contribute to the understanding of Cd^2+^ toxicity mechanisms. The group that developed the ZinCh sensor also developed a Cd^2+^ sensor from ZinCh by modifying the cysteine residues to avoid the binding of Zn^2+^ to histidine, which resulted in enhanced selectivity for Cd^2+^ (Vinkenborg et al. 2011). The double mutations of N144C/A206C, A206C/S208C, or Q204C/A206C into both FPs in a CFP–YFP improved the Cd^2+^ affinity from approximately 80 nM for the ZinCh to 15, 0.1, and 2.4 nM of *K*_d_[Cd^2+^], respectively. These Cd^2+^ sensors also sensed the other metal ions showing similar coordination chemistry, such as Co^2+^ and possibly Ni^2+^, although they specifically detected change in the Cd^2+^ concentration in the presence of high concentrations of other metal ions (Fe^3+^, Cu^2+^, Ba^2+^, Mg^2+^, or Ca^2+^), including Zn^2+^. Although further improvements in sensors are desirable in terms of intracellular Cd^2+^ measurements, the development of GE Cd^2+^ sensors is expected to be a stepping stone in investigations aimed at assessing Cd^2+^ toxicity to humans in the future.

Control of the selectivity of the intrinsic ion dependency of FPs is another strategy used to develop ion sensors. As mentioned above, *Aequorea* GFP variants, including the T203Y mutation, are Cl^-^ sensitive (Wachter and Remington 1999; Griesbeck et al. 2001; Arosio et al. 2007; ). A Ca^2+^GE indicator was developed by engineering a Ca^2+^ binding site in the EGFP (Tang et al. 2011). To this end, the authors created five variants by introducing charged residues (glutamic acid or aspartic acid) at specific substitution sites, namely, S147, S202, Q204, F223, and T225. The authors state that the precise geometric properties generated by these substitutions are responsible for a Ca^2+^ chelation near the chromophore. Based on the same strategy, Koldenkova and colleagues screened the mutations in cpVenus to selectively sense Mg^2+^ or Ca^2+^ ions and successfully found the right mutations (Koldenkova et al. 2015). While the intracellular concentration of Mg^2+^ in mammalian cells is in the range of 15–20 mM, Ca^2+^ concentrations are <1 mM. Thus, the ratiometric [Mg^2+^]/[Ca^2+^]-sensitive cpVenus has only a small response to the cytosolic changes in Ca^2+^, which in turn allows it to monitor selectively the variations in Mg^2+^ concentrations. The affinities of cpVenus for Mg^2+^ and Ca^2+^ are *K*_d_[Mg^2+^] = 5.1 mM and *K*_d_[Ca^2+^] = 4.8 mM, respectively. One interesting point in the development of this sensor was the need to avoid the FRET effect in order to make the ratiometry calculation simpler. Moreover, the authors fused the [Mg^2+^]/[Ca^2+^]-sensitive cpVenus to a red FP which is insensitive to [Mg^2+^]/[Ca^2+^]. This newly developed GE Mg^2+^ sensor was named MagIC. Although the development strategy of the MagIC may look similar to that of other FRET sensors, its concept is quite different as the authors removed the FRET effect and attempted to control ion selectivity.

A FP-based nitric oxide (NO) sensor was developed by utilizing a previously developed cGMP FRET sensor named CGY (Sato et al. 2005b, 2006). These authors fused a soluble guanylate cyclase that produces cGMP from GTP to CGY so that the CGY could immediately sense the NO produced by the guanylate cyclase. The soluble guanylate cyclase is a heterodimer composed of α- and β-subunits, and the heterodimerization spontaneously occurs but depends on NO binding. This NO sensor was constructed by fusing a pair of subunits with a CGY. The concentration of cGMP locally increases around this NO sensor, with the increase in the heterodimer population depending on the NO concentration, and then the CGY sensors sense the produced cGMP. Although the expression level of the NO sensor in cells must be sufficiently low so that the produced cGMP does not affect cell behavior, this development strategy is quite unique and may serve as a useful reference for the future developments of other GE sensors.

In the last part of this section, we introduce one final interesting example of the development strategy associated with GE sensors which involves the use of a light-oxygen-voltage (LOV) sensing domain instead of conventional FPs (Buckley et al. 2015). The LOV domain has a binding motif to flavin mononucleotide, and the conformation of the LOV domain changes with blue light illumination via a covalent linkage between the flavin cofactor and the thiol moiety in the LOV (Harper et al. 2003). The unfolding of the α-helix is responsible for the light-dependent conformational change, and this dynamic conformational change can be utilized to control the activity of proteins (e.g., the catalytic surface is veiled or unveiled depending on the conformational change). The LOV domain has been used as a tool for optogenetics, which is the combination of genetics and optics, to control cellular events (Pudasaini et al. 2015). In addition, because the flavin also acts as a chromophore, the LOV domain has been used as an alternative to FPs (Buckley et al. 2015). While the fluorescence of the LOV domain is quite low, it presents several advantages, such as it's the smaller size (approx. 10 kDa) by comparison with the size of FPs (approx. 25 kDa), along with its pH insensitivity and thermal stability. Chapman and colleagues performed DNA shuffling of a phototropin LOV domain to increase the fluorescence intensity, constructing a LOV domain that was named iLOV (Chapman et al. 2008). The iLOV emits a green fluorescence with a peak wavelength of 495 nm when excited with 440- to 460-nm light. The fluorescence of the iLOV was found to be quenched by the addition of metal ions in solution. Especially, copper ions (Cu^2+^) dramatically decreased the iLOV fluorescence in comparison to that of other ions (Ravikumar et al. 2015). Therefore, LOV can act as a Cu^2+^ sensor. While the iLOV requires the external supply of a substrate, the turnover of substrate prevents the photobleaching, which is another strong advantage of the iLOV. In future, novel mutations might make iLOV sensitive to various ions by taking advantage of the development strategies of the current GE sensors.

## Redox indicators

Redox reactions are involved in various biochemical processes, such as cell signaling, regulation of transcription, oxidative phosphorylation, photosynthesis, and/or the catabolic reactions during cellular respiration. The production of H_2_O_2_, in particular, is currently considered to be a good redox indicator and was used as a target for GE sensor development. For example, GE H_2_O_2_ sensors greatly facilitate the investigation of immune responses when whole body imaging is required. However, the development strategy for such sensors is quite complicated because the oxidization status affects not only the chromophore environment, but also the protein structure, and the convolution of all of these effects affects the output fluorescence. There are currently three well-known FP-based H_2_O_2_ sensors: HyPer (Belousov et al. 2006), rxRFP (Fan et al. 2015), and roGFP (Dooley et al. 2004; Hanson et al. 2004).

The HyPer and rxRFP sensors were developed based on the Pericam- or GECO- like strategy. For the development of HyPer, the regulatory domain of the prokaryotic H_2_O_2_-sensing protein (OxyR-RD) was inserted into a cpYFP (Belousov et al. 2006). The red color variant of this sensor, HyPerRed, was developed by replacing the CaM and the M13 peptide in the R-GECO with the OxyR-RD because the red color is an advantage in deep tissue imaging or whole mouse imaging (Ermakova et al. 2014). In the rxRFP, the two peptides, including several cysteine residues, were used instead of the OxyR-RD in the HyPerRed (Fan et al. 2015). These two peptides were designed so as to be annealed via the disulfide bond of cysteine–cysteine. In its oxidized state, the red cpFP (mApple in the case of rxRFP) emits fluorescence because the annealing of the two peptides stabilizes the structure of the cpFP. The deoxidation of the disulfide bond causes the structure of the cpFP to become disordered, resulting in the loss of fluorescence. This notion of using a pair of artificial H_2_O_2_-sensing peptides was also applied to a FRET redox indicator (Kolossov et al. 2008, 2011). Kolossov and coworkers developed redox-sensitive peptides, referred to as a redox linker (RL), which were composed of continuous α-helical sequences (EAAAK) with cysteine residues and then used these peptides as a linker into a CFP–YFP pair (Kolossov et al. 2008). The RL formed a “clamped-coil” structure via the disulfide conjugations in an oxidized state but formed a long α-helical structure in a deoxidized situation. Hence, the distance between the CFP and the YFP was dependent on the oxidization state. These authors engineers a number of linkers, of which the best was named RL7 [ESPC(EAAAK)_3_CESSSKC(EAAAK)_4_CEF]. The probe of CFP–RL7–YFP, named CY–RL7, exhibited a higher midpoint redox potential than that of the previous redox sensor and is thought to be suitable for measuring glutathione redox potentials in mammalian cells.

The development strategy of rxYFP was based on a different approach than that for rxRFP, although the names are similar (Ostergaard et al. 2001). The development strategy was as follows. Basically, the three consecutive β-strands, i.e., 7, 10, and 11, prevent the chromophore of the *Aequorea* GFP from interacting with the solvent (Heim and Tsien 1996; Ormö et al. 1996; Yang et al. 1996; Phillips 1997). If two residues of adjacent β-strands are substituted with cysteine so that the two β-strands are conjugated by the disulfide bond in oxidized situation, the conjugation of the two β-strands causes the structural distortion of the β-barrel structure. This distortion allows water to invade the β-barrel structure, with the result that the fluorescence intensity of the substituted FP decreases due to the interaction of water molecules with the chromophore in the oxidized solution. Then, when the two adjacent β-sheets become separated by deoxidization of the disulfide bond in the redox state, the fluorescence intensity is recovered with the re-formation of the original β-barrel structure. Thus, this substituted FP is able to monitor the compartment-specific redox variations caused by H_2_O_2_. The candidate of the substitutions included His-148th, Tyr-203th, Glu-222th, Asn-146th, and Ser-205th, all of which are residues interacting with the chromophore (Ehrig et al. 1995; Heim and Tsien 1996; Ormö et al. 1996). Specifically, four mutation pairs were tested in YFP: S147C/Q204C, N149C/S202C, S202C/T225C, and Q204C/F223C (Ostergaard et al. 2001). While YFP–Q204C/F223C unfortunately formed a dimer during oxidation, the other three pairs retained a monomeric state. The YFP–N149C/S202C construct (named rxYFP) showed the most obvious changes in terms of fluorescence intensities between the oxidized and deoxidized situations among the mutants. However, this construct was found to be greatly affected by pH and low concentrations of metal anions and did not show the spectral changes necessary for ratiometic measurements.

The roGFPs were developed based on the same strategy as that used for rxYFP to confer the ability of ratiometric measurement (Dooley et al. 2004; Hanson et al. 2004). Because the wild-type *Aequorea* GFP had two cysteine residues at Cys-48th and Cys-70th, cysteine-free GFP variants were developed to maximize the effectivity of the disulfide reactions after cysteine substitution for β-strand conjugation. It was then determined that the substitution of the Cys-70th deleteriously affected the solubility of the GFP, so the GFP–C48S mutant was used as a template for development. At that time, the GFP that was widely used was the original wild-type *Aequorea* GFP, which included a trivial mutation, Q80R, thought to be caused by PCR error (Tsien 1998). To make the GFP–C48S/Q80R indicative of redox state, Hanson et al. (2004) tested the two mutation pairs and various combinations (S147C/Q204C, N149C/S202C, and S147C/Q204C/N149C/S202C). The resulting roGFP1 was obtained from the GFP–C48S/Q80R/S147C/Q204C construct, and roGFP2 was subsequently obtained by the substitution of S65T. The excitation spectra of roGFP1 and roGFP2 show two major peaks at approximately 395 nm and 490 nm, and the peak intensities differ between the two roGFPs. The sensitivity of the roGFP2 was improved by substituting Phe-223th and Ala-206th, which are close to the key cysteine residue (Q204C), into lysine residues (Dooley et al. 2004). These authors suggested that the positive charges of the lysine residue placed near cysteine residues increased the sensitivity. The midpoint potentials of roGFP1 and roGFP2 are −287 and −272 mV, respectively. These probes are therefore suited for use in mitochondria or cytoplasm, whose estimated reduction potential is about 350 mV— but not in the endoplasmic reticulum, whose redox potential is approximately 180 mV. In later development stages, the insertion of an amino acid altered the thermodynamic stability of the disulfide bond in roGFP1, and the midpoint redox potential could be shifted by inserting an amino acid behind Cys-147th in roGFP1, which was named roGFP1-iX where X denotes the inserted amino acid (Lohman and Remington 2008). For example, the modified glutamate-inserted roGFP1 (roGFP1-Ie probe) showed −236 mV of reduction potential and is better suited for observational studies in more oxidizing subcellular compartments than the cytoplasm.

An FP-based redox indicator based on the Cameleon strategy, named Redoxfluor, has been developed recently (Yano et al. 2010). Redoxfluor is composed of Cerulean, a cyan variant of *Aequorea* (Rizzo et al. 2004), Citrene, and a yeast carboxy-terminal cysteine-rich domain (c-CRD) (Kuge et al. 1998) tandemly inserted between Cerulean and Citrene. The redox state induces the conformational change of the c-CRDs, resulting in the spectral change of the Cerulean/Citrene complex though FRET. Although the small degree of change is one of the remaining problems of current H_2_O_2_ sensors, a similar tandem Redox sensor named Oba-Q showed improved performance in terms of detecting the redox state, although ratiometric measurements are not yet possible: the quantum yield of Oba-Q in an oxidized situation was found to be approximately sixfold higher than that in the deoxidized situation. Oba-Q consists of a pair of blue and cyan variants of *Aequorea* GFP; as such, Oba-Q is the first blue-colored GE redox sensor (Sugiura et al. 2015). From an historical point of view, the development of the GE redox sensors is more recent than those of ion-sensitive FPs. Thus, it is expected that further developments and improvements will resolve the remaining issues of the current sensors, such as the non-specificity or the sensitivity depending on experimental conditions, which strongly limits the possibility for precise quantitative measurements.

## Fluorescent GE sensors of membrane potential

The use of electrophysiological methods to measure membrane potential has a long history. In past decades, organic dyes have been developed to measure membrane potentials, both in single cells (e.g., to study the physiology of single neurons) and in large populations of cells. However, in spite of the high signal-to-noise ratio and even the possibility for single cell resolution, the critical problem of invasiness remained critical. At present, GE voltage sensors represent the best low-invasive method for optical microscopy and offer high spatial and temporal resolution for in vivo imaging.

The first GE voltage sensor used to measure membrane potential was developed in 1997 and was named FlaSh, which stands for Fluorescent Shaker (Siegel and Isacoff 1997). FlaSh is a fusion protein in which the natural GFP was modified to harbor a deletion at 233th–238th at the C-terminus, and then was inserted into a voltage-activated Shaker K^+^ channel (originating from *Drosophila*) at a site located just after the sixth transmembrane domain (Tempel et al. 1987). The developers of FlaSh expected that (1) the electro-environment or the conformational change of the channel would affect the fluorescence intensity and/or spectrum of the fused GFP and (2) that the deletion of six residues at the C-terminus of the GFP would improve the on/off contrast of the fluorescence. While the GFP deletion mutant alone does not emit fluorescence, the FlaSh reports changes in membrane potential due to the voltage-dependent rearrangements occurring in the K^+^ channel. The first version of FlaSh could detect only slow reactions (τ_on_ = ~100 ms). However, 5 years later, an improved version of FlaSh was successfully used to detect fast responses (τ_on_ = ~5 ms) by replacing the GFP with an ecliptic variant of GFP (GFP–S147D/N149Q/T161I/S202F/Q204T/A206T) (Guerrero et al. 2002). In order to enhance the effect of the chromophore environment on the membrane potential or on the conformational change of the channel, Jin and colleagues divided the Venus FP into two parts and randomly inserted each half into Shaker monomers via a transposon reaction (Jin et al. 2011). Since Shaker dimerizes on the membrane, the separated Venus parts that completely lacked fluorescence could bind to each other and form the original structure so as to emit fluorescence. These authors tested 120 recombinants of the split-site in Venus fused to Shaker and reported success in improving the response time of FlaSh by sixfold (τ_on_ = ~15 ms). They therefore demonstrated for the first time that the electrical activity in excitable cells could be studied through the genetic introduction of fluorescent probes.

Following this work, other probes were developed based on the same development strategy. SPARC (sodium channel protein-based activity reporting construct), not to be confounded with SPARC (secreted protein acidic and rich in cysteine), is an engineered optical channel-gating reporter (Ataka and Pieribone 2002). To develop the SPARC sensor, an enhanced GFP (GFP including the F64L/S65T mutation) was inserted into the intracellular loop between the second and third domain of a rat skeletal muscle (μ1) sodium channel (Isacoff et al. 1990). Thus, while FlaSh is K^+^ sensitive, SPARC reports the gating of the sodium channel. Another group developed a sensor for membrane potential named VSFP1 (Sakai et al. 2001). VSFP1 utilized the first to fourth transmembrane segments among six transmembrane segments of a rat voltage-gated K^+^ channel called the Kv2.1 channel. The fourth segment in the Kv2.1 channel was expected to rotate dynamically with membrane depolarization (Cha et al. 1999). A pair of CFP and YFP conjugated with a single amino acid linker was fused to the end of the fourth segment of the Kv2.1. The voltage-dependent conformational change of the fourth segment would alter the arrangement of the dipole moments of CFP and YFP, causing the spectral change of the probe. Thus, VSFP1 is able to follow changes in transmembrane potential with a high sensitivity.

Following the engineering of FlaSh, SPARC, and VSFP1, later developments of voltage-sensitive sensors were dramatically changed by the finding of *Ciona intestinalis* voltage-sensing phosphatase (Ci-VSP), a homolog to the voltage sensor domain of the Kv2.1 channel (Murata et al. 2005). Surprisingly, Ci-VSP is not an ion channel, but a phosphatase whose structure resembles the Kv2.1 voltage sensor domain. Ci-VSP can respond to variations of membrane potential even when freed from its catalytic domain. The Kv2.1 voltage sensor domain in VSFP1 was then replaced by Ci-VSP without its catalytic domain. The fourth segment of the modified Ci-VSP was mutated to improve the voltage sensitivity, and a new sensor, named VSFP2.1, was engineered and described as a fast membrane potential sensor (Dimitrov et al. 2007). VSFP2.1 was used in the first optical observational study which reported action potentials in neuron-like PC12 cells. Because Ci-VSP functions as a monomer on the plasma membrane (Kohout et al. 2008), another advantage of its use is that the VSFP2.1 can smoothly localize to the plasma membrane as oligomerization on the endoplasmic reticulum is not required.

The improvement in VSFPs has continued since. The peptide linker between Ci-VSP and the CFP–YFP was further optimized (VSFP2.3) (Lundby et al. 2008, 2010), and red variants of VSFP2.1 were also developed, such as Mermaid (Tsutsui et al. 2008), VSFP2.4 (Mutoh et al. 2009), and VSFP-CR (Lam et al. 2012). To improve the FRET response within the VSFP series, the donor and the acceptor of a FRET pair were respectively fused to the N- and C-terminus of a Ci-VSP, similar to Cameleon (Akemann et al. 2012). This minor change induced the shift of the dynamic range to a more negative potential while maintaining the fast kinetics of voltage sensing. Furthermore, it was found that the chimera of Ci-VSP and homologous portions of the Kv3.1 voltage-gated potassium channel subunit could shorten the limited response kinetics of the VSFPs (Mishina et al. 2012, 2014). Other voltage-sensing phosphatases derived from *Nematostella vectensis* and *Danio rerio* were also tested for their application in the construction of VSFP-like voltage sensors (Baker et al. 2012).

Single FP versions of VSFP2.1 have been also developed with (Gautam et al. 2009; Barnett et al. 2012) or without circular-permutation (Perron et al. 2009; Jin et al. 2012). For example, ArcLight is a series of probes originally designed to monitor neuronal electrical activity. The ArcLight FP was designed from the derivative of *Aequorea* GFP and termed ecliptic pHluorin (Miesenböck et al. 1998). A spontaneous mutant, A227D, exhibited significantly improved response amplitudes while maintaining the relatively high-speed response (τ_on_ = ~10 ms) (Jin et al. 2012). Among the numerous other VSFP-like sensors, the one showing the highest performance is ASAP1 (i.e., Accelerated Sensor of Action Potentials 1). The response time (τ_on_) of ASAP1 has been reported to reach 2 ms (St-Pierre et al. 2014). The ASAP sensor used a voltage-sensing phosphatase derived from *Gallus gallus* instead of Ci-VSP, and the FP was placed not inside but outside of the cell. Overall, it might be no exaggeration to say that the concept of the FlaSh development established the foundation of the GE voltage sensors to measure membrane potential, and that of the VSFP series greatly contributed to improved applicability of these sensors to measure the fast reaction of the membrane action potentials.

Another distinct category of GE membrane sensors is based on the use of proteorhodopsin, a light-driven proton pump found in marine planktonic bacteria, Archaea, and eukaryotes (Gautier et al. 2012; Gong 2015). The proteorhodopsin is a natural GE sensor of membrane potential, and it has been commonly used as an optogenetic tool, similarly as the LOV domain mentioned above (Gautier et al. 2012; Pudasaini et al. 2015). Rhodopsin is a pigment found in photoreceptors of retinal cells whose chromophores have photocharacteristics determined by the protonation states of the Shiff base of the aldehyde in the retina. In the case of a green-absorbing proteorhodopsin (GPR), derived from *Halobacterium salinarum*, the chromophore emits fluorescence in the protonated state but not in the deprotonated state. The first proteorhodopsin optical proton sensor (PROPS) was developed by optimizing the pKa of the GPR with a single amino acid mutation (Kralj et al. 2011). Even though PROPS actually enable the measurement of electrical spiking in *Escherichia coli* to be measured, the first PROPS could not be applied in mammalian neurons because of its inability to localize to the eukaryotic plasma membrane. However, PROPS has a high photostability, similar to that of iLOV, due to the turnover of the retina.

Archaerhodopsin 3 (Arch), which is a proteorhodopsin derived from *Halorubrum sodomense*, allowed researchers to observe electrical spiking in mammalian cells (Kralj et al. 2012). While the photostability of Arch was lower than that of FPs, the response time (τ_on_) of 0.5 ms is one of the fastest among the recent GE voltage sensors to measure membrane potentials. The fluorescence of Arch induces proton pumping on its own, causing a background photo-current of 10–20 pA. A mutation of D95N in Arch was found to diminish the self-proton pumping and improve its dynamic range by threefold but to degrade the response time by eightfold (τ_on_ = ~40 ms) (Kralj et al. 2012). This slow response was subsequently improved with further mutations to finally reach a τ_on_ value of approximately 1.0 ms (Gong et al. 2013). Two additional mutants, QuasAr1, and QuasAr2, which exhibit a high brightness, high-speed response and non-pumping characteristics, were obtained by screening a library of >10^4^ Arch variants using random mutagenesis techniques (Hochbaum et al. 2014). The improvement in the brightness and response speed, in particular, was remarkable. By comparison to the wild-type Arch, QuasAr1 was found to be 15-fold brighter and tenfold faster (τ_on_ = ~50 μs), and QuasAr2 to be threefold brighter and twofold slower (τ_on_ = ~1 ms). The response amplitude of QuasAr2 was 1.5-fold larger than that of the wild-type Arch, although this characteristic was not improved in QuasAr1. In short, compared with QuasAr2, QuasAr1 is better in terms of brightness and response speed, but it has a lower dynamic range (i.e., response amplitude).

Arch-like voltage sensors for membrane potentials could have become more popular if not for the critical issue of the lower quantum yield of Arch (9 × 10^−4^) by comparison to the wild-type GFP (0.79). This problem proved to be surmountable by using an energy transfer phenomenon (Zou et al. 2014). The strategy was to place a FP with a high-quantum yield close to an Arch-type probe, so that the excited energy of the FP could be transferred to the retinal chromophore. Doing so, the observer could record the fluorescence intensity emitted by the FP rather than from the retinal sensor. This strategy provided not only to solve the problem of the low quantum yield of Arch sensors, but it also provided the possibility to develop color variants of voltage sensors. The same notion was independently realized using another kind of rhodopsin derived from *Leptosphaeria maculans* (Mac) because of its fast kinetics (Gong et al. 2014). Mutations were added into the Mac rhodopsin to improve the photo-response and avoid the steady state of the photo-current production. The mutated Mac that showed the best performance was named MacQ and was fused later to Citrene or mOrange2. The MacQ sensors exhibit a fast response (τ_on_ = ~3.0 ms) and enabled not only neural spiking and sub-threshold dynamics of membrane potential in pyramidal neurons to be monitored, but also calcium spike-induced regenerative potentials of the dendrites of Purkinje neurons in living mice. Thanks to these achievements, the Arch-type sensors have become the new generation of GE voltage sensors.

## Sensing temperature, pressure, or molecular crowding in living cells

### Temperature

Water plays an essential role in the function of biological macromolecules, proteins, and DNA of all living organisms. The motility of water molecules affects protein functions, including protein-folding, enzymatic activity, among other processes. The motility of water molecules depends directly on the temperature of the solution. There are only a limited number of GE probes which are applicable for thermosensing or pressure sensing.

Upon transfecting a temperature-sensitive expression vector coding any kind of fluorophore into a cell, the temperature of the cell expressing this system can be estimated from the fluorescence—even in a whole cell. The actual testing of this concept was performed in *Escherichia coli* using β-galactosidase, which emits green fluorescence, as a temperature reporter (McCabe et al. 2011). This strategy was thought useful to monitor the temperature at single cell resolution in whole brain or at the whole body level to monitor, for example, the generation of fever during the immune response. However, this method cannot map the intracellular temperature localization nor can it detect fast temperature responses.

Cell temperatures can also be measured by utilizing the temperature dependency of the fluorescence polarization anisotropy of fluorescent dyes (i.e., Brownian rotational motion of the fluorophores depends on the temperature). Hence, intracellular temperature mapping was realized by anisotropy measurement of the FP (Donner et al. 2012). Specifically, the temperature changes of single neurons in *Caenorhabditis elegans* could be observed, and this marked the first actual application of the GE thermosensor in a model animal (Donner et al. 2013). Importantly, the anisotropy does not depend on pH and metal ions, but is affected by viscosity. This approach is expected to be highly applicable at various scales, from single cell to whole body imaging, under an appropriate microscopic setup for the measurement of fluorescent anisotropy.

In an effort to visualize intracellular thermal changes in living cells, a temperature-sensitive GFP called tsGFP was developed based on a thermosensitive coiled-coil protein (Hurme et al. 1997). The tsGFP was built using a thermosensitive coiled-coil protein TlpA, derived from *Salmonella*. The fluorophore-forming region of the *Aequorea* GFP was inserted between tandem repeats of the coiled–coil region of TlpA or of the full-length TlpA. The authors took advantage of the fact that the *Aequorea* GFP has two excitation peaks at approximately 400 nm and 480 nm, respectively, which correspond to the phenolic and phenolate forms of the chromophore. The phenolic–phenolate transition is quite sensitive to the condition of the β-barrel structure. Because the wild-type GFP weakly dimerizes in solution when the two GFPs are brought into proximity, the ratio of the excitation efficiency at approximately 400 nm and 480 nm changes due to small structural changes in the β-barrel caused by the dimerization (Kiyonaka et al. 2013). As a result, the dimerization of the tsGFP converts temperature changes into observable fluorescence changes. While the shape of the emission spectrum of GFP itself is intrinsically sensitive to the temperature, the fused TlpA fragment enhances the spectral change in the range of 35–40 °C. In future studies, the temperature sensitivity and the dynamic range of tsGFP are expected to be improved by using strategies developed for other kinds of sensors, as mentioned above.

### Pressure

**Fig. 3:**
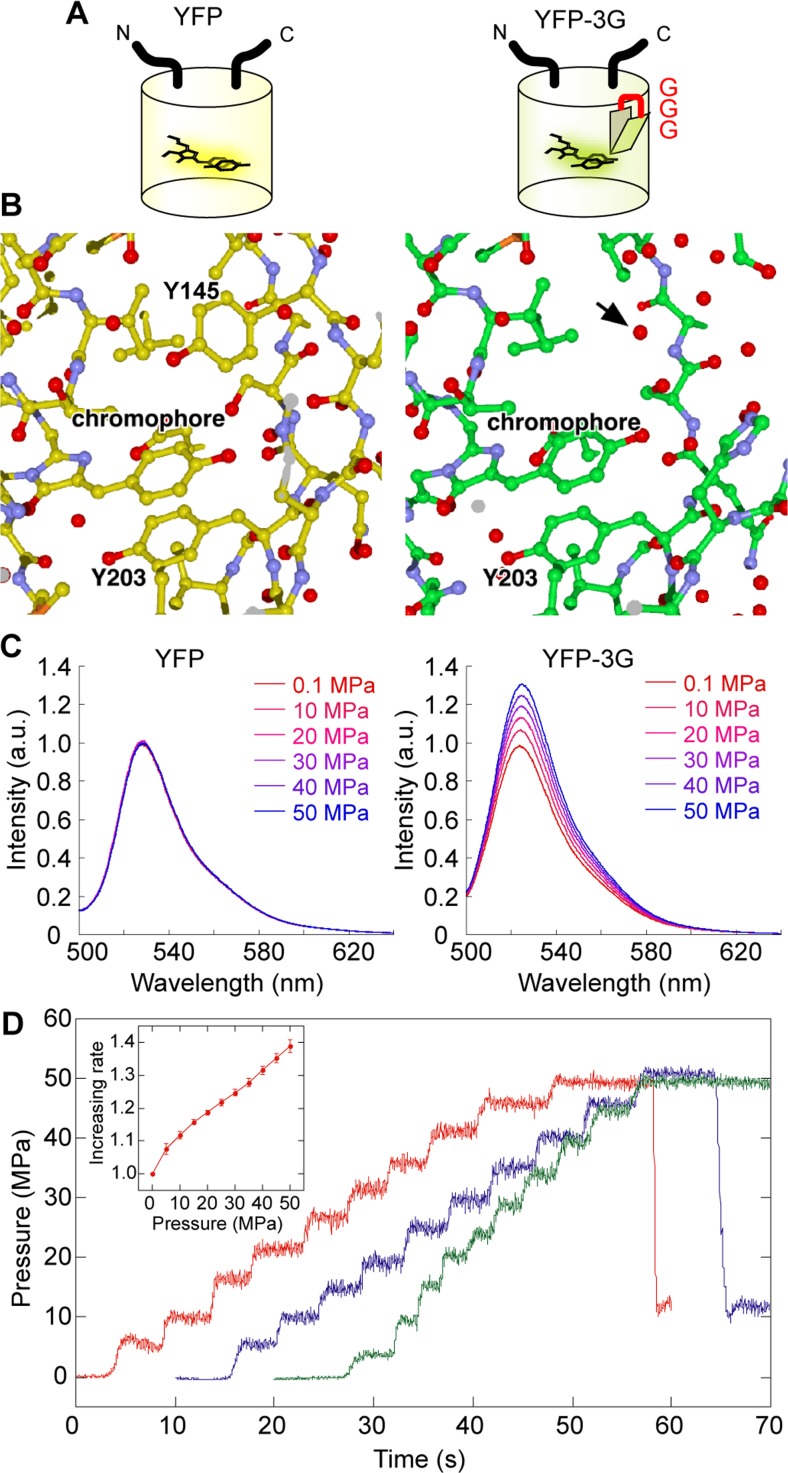
Yellow fluorescent protein (YFP) and a pressure-sensitive yellow fluorescent protein variant (*YFP-3G*). **a** Schematic drawing of YFP and YFP-3G. **b** Crystal structure of YFP (*left*) and YFP-inserted ‘GGG’ (*right*). *Arrow* indicates the oxygens included in water molecules filling the space of YFP’s Tyr145. (**c**) Pressure-dependant fluorescence of YFP (*left*) and YFP-3G (*right*) at 0.1 (*red*) to 50 (*blue*) MPa. All spectra are normalized with the spectrum at 0.1 MPa. The traces represent the average of six individual trials. **d** Time-course changes in fluorescence intensity measured at 515–535 nm with increasing hydrostatic pressure at steps of 5 MPa. *Different colors* indicate different trials. *Insert* Calibration table showing the changes in fluorescence and hydrostatic pressure as a ratio

Pressure is another parameter responsible for the water state which is involved in many biological behaviors, including osmotic pressure, bleb-based motility (e.g., of some cancer cells or even parasites), membrane stress, blood pressure, among others. The pressure levels exerted on the cell membrane can be directly measured by using a contact probe, such as in the atomic force microscopy. For the first time, we developed a GE pressure sensor (Watanabe et al. 2013) based on the notion that the fluorescence of FPs is affected by the pressure of the solution (Verkhusha et al. 2003; McAnaney et al. 2005; Mauring et al. 2005; Barstow et al. 2008). We engineered a YFP variant with enhanced pressure dependency by inserting three glycine residues just before the β-strand 7 (Try-145th) (Fig. 3). According to the crystal structure analysis, the three glycine (3G) insertion produced a small space for water molecules to locate statically near the chromophore (Fig. 3b). This static water was thought to quench the fluorescence and was discharged to the outside of the β-barrel at high pressure. While the fluorescence intensity of the YFP sensor changed only slightly with pressure increase in the range of 0.1–50 MPa, that of the 3G-inserted YFP increased and its sensitivity was enhanced (Fig. 3c). Although the precise mechanism has not yet been clarified, we successfully obtained time-course images of the pressure changes of the solution (Fig. 3d) and measured pressure variations in living *Escherichia coli*. cells under pressures ranging from 0 to 150 MPa (Watanabe et al. 2013).

### Molecular crowding

As described in Goodsell's “The machinery of life”, a cell is more densely populated with macromolecules and proteins than with water molecules (Goodsell 2009). The molecular crowding conditions, such as protein density, directly affects protein assembly, regulation, and activity. Thus, the density of the crowding agent, i.e., the protein concentration in cells, is a valuable factor for evaluating intracellular crowding if it can be measured separately from the diffusion of fluorescent dyes, nanoparticles, or proteins (van den Berg et al. 1999). A macromolecular crowding FRET sensor was recently developed based on the Cameleon-like strategy (Boersma et al. 2015). The protein inserted between a Cerulean and a Citrine pair formed an artificially designed hinge structure domain composed of two α-helical peptides of A(EAAAK)_6_A and the flexible linker of (GSG)_6_ (this hinge-like domain was inserted into the FRET pair with the same flexible linkers). This α-helical peptide is quite similar to the RL peptide in the redox sensor CY-RL7, but does not include cysteine residues. The hinge-like domain formed an opened- and condensed-conformation depending on the crowded condition due to the excluded-volume effect. The transition of the opened and condensed conformation reflected FRET efficiency. Furthermore, we proposed another strategy to enhance the crowded sensitivity of YFP (Morikawa et al. 2016). In the enhancement of the pressure dependency of YFP, one glycine insertion made the YFP sensitive to hydrophobicity in solution, while the wild-type YFP and CFP did not exhibit this sensitivity. The FRET pair of CFP and the glycine-inserted YFP yielded a GE crowding sensor which successfully detected the changes in protein concentrations during the cell division or when forcibly provoking the swelling or shrinking of the cell. These studies showed that the development of GE temperature, pressure, and crowding sensors are just in their starting gate and that many original strategies and improvements are to be expected.

## Conclusion

Since the first isolation of the *Aequorea* GFP, many GE sensors have been developed to monitor intracellular chemical/physical conditions with the aim to elucidate various biological phenomena. Often, the FP-based sensors have a shared history of concepts and development strategies, namely, enhancing the intrinsic sensitivity, utilizing the sensing domain of other proteins, or the pairing of those phenomena and of various fluorescence characteristics.

Undoubtedly, FP-based GE sensors will continue to be developed in the future and will contribute to the discovery and analysis if new biological behaviors. The development tools, such as a cell-free protein synthesis system, may help in the development of new GE sensors. The development of new types of GE sensors that can distinguish a single parameter is strongly desirable. For example, the alternatives to conventional FPs, such as iLOV and fluorescent opsin, are likely to bring new development possibilities due to their small size and high photostability. Not only will GE sensors provide new biological information, but they will also open the door to brilliant inspirations in future developments.

In this review, we have focused on the GE sensors engineered to measure the intracellular conditions of cells. However, another interesting aspect would be to measure physical parameters, such as strain/tension or force, as mechanical stimuli are known to influence biological behaviors and biochemistry. Interestingly, strain/tension sensors have been recently developed based on the combination of circular permutation, FRET, or protein proximity imaging (PRIM) (Iwai and Uyeda 2008; Meng et al. 2008; Grashoff et al. 2010; Meng and Sachs 2011; Ichimura et al. 2012). There are many other important mechanical parameters, including elasticity, viscosity, shear stress, that require the development of mechanical sensors. The basic development strategy for these sensors is likely to use similar strategies to those ones described in this review. However, the efforts required to develop sensors for these kinds of parameters are expected to be more challenging because the chemical/physical parameters are more or less related to each other in most biological cases.
